# Supplementary material for: Synthesis and Antineoplastic Activity of a Dimer, Spiroindolinone Pyrrolidinecarboxamide
Source: Molecules. 2023 May 5;28(9):3912. doi: 10.3390/molecules28093912 (PMC10180320; doi:10.3390/molecules28093912)
Supplement: Supplementary file 1 [file molecules-28-03912-s001.zip › molecules-2330352-supplementary/HRMS data of XR-4.pdf]

1

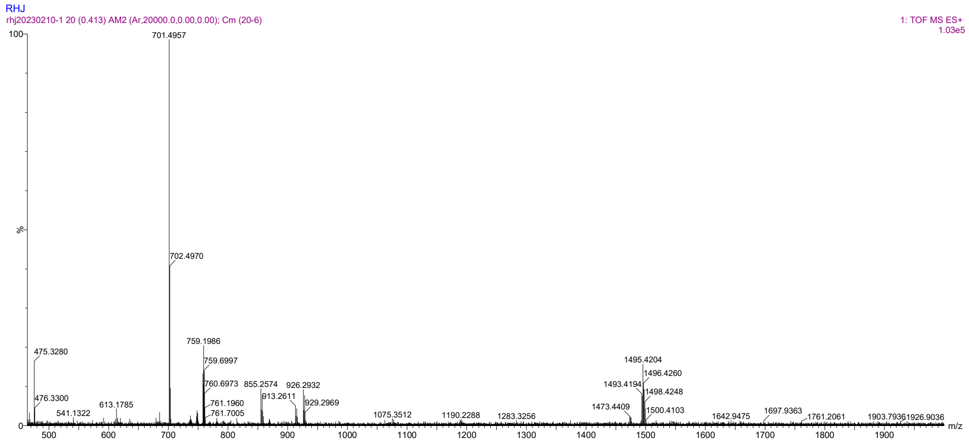

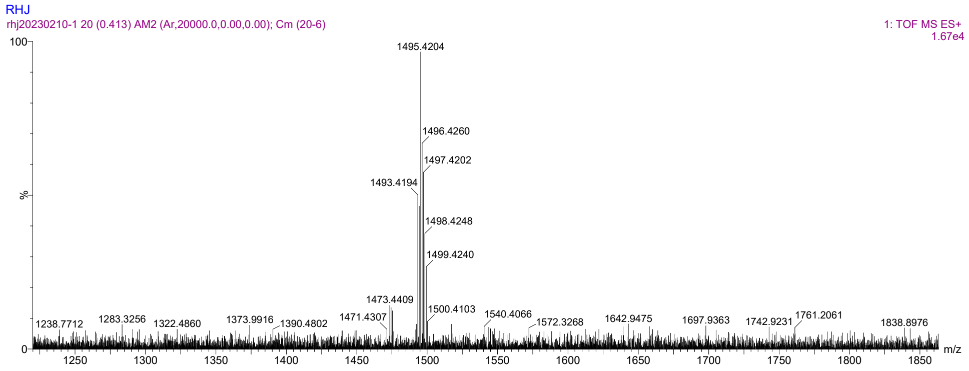

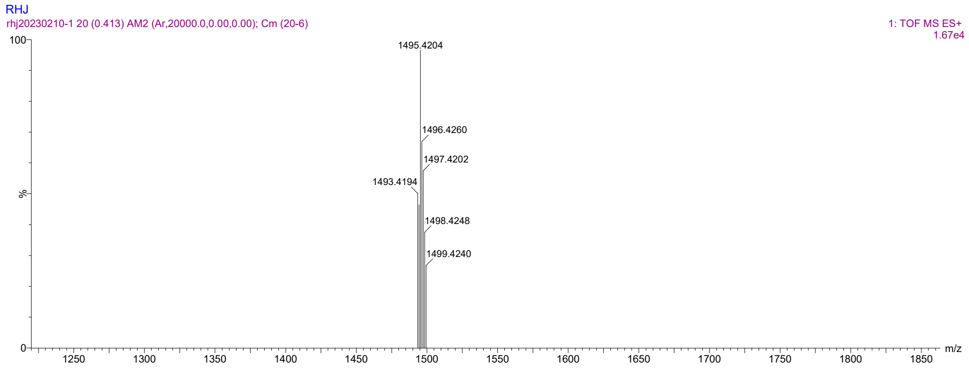

Elemental Composition Report

Single Mass Analysis

Tolerance = 1.0 mDa / DBE: min = -1.5, max = 100.0

Element prediction: Off

Number of isotope peaks used for i-FIT = 9

Monoisotopic Mass, Even Electron Ions

1983 formula(e) evaluated with 1 results within limits (up to 50 closest results for each mass)

Elements Used:

C: 0-100   H: 0-200   N: 0-8   O: 0-15   F: 2-2   Na: 1-1   Cl: 4-4

Mass	Calc. Mass	mDa	PPM	DBE	i-FIT	Norm	Conf(%)	Formula
Minimum:				-1.5				
Maximum:		1.0	10.0	100.0				
1495.4204	1495.4207	-0.3	-0.2	34.5	568.8	n/a	n/a	C72 H78 N8 O15 F2 Na Cl4
