# Supplementary figures and images for: Synthesis and Antineoplastic Activity of a Dimer, Spiroindolinone Pyrrolidinecarboxamide
Source: Molecules. 2023 May 5;28(9):3912. doi: 10.3390/molecules28093912 (PMC10180320; doi:10.3390/molecules28093912)

rhj-3-61-cdc13+c

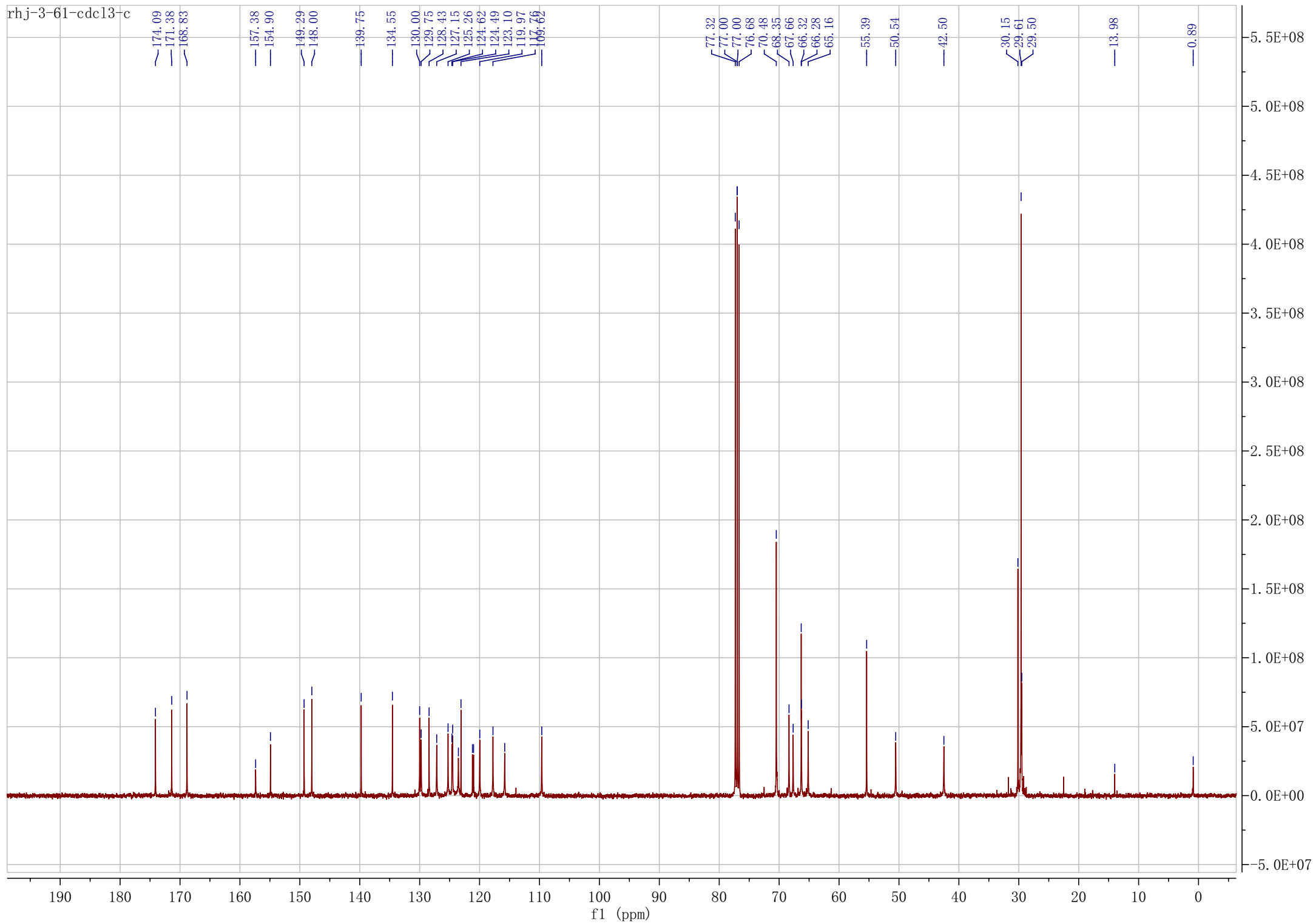

Supplement: Supplementary file 1 [file molecules-28-03912-s001.zip › molecules-2330352-supplementary/CNMR data of XR-4.pdf]

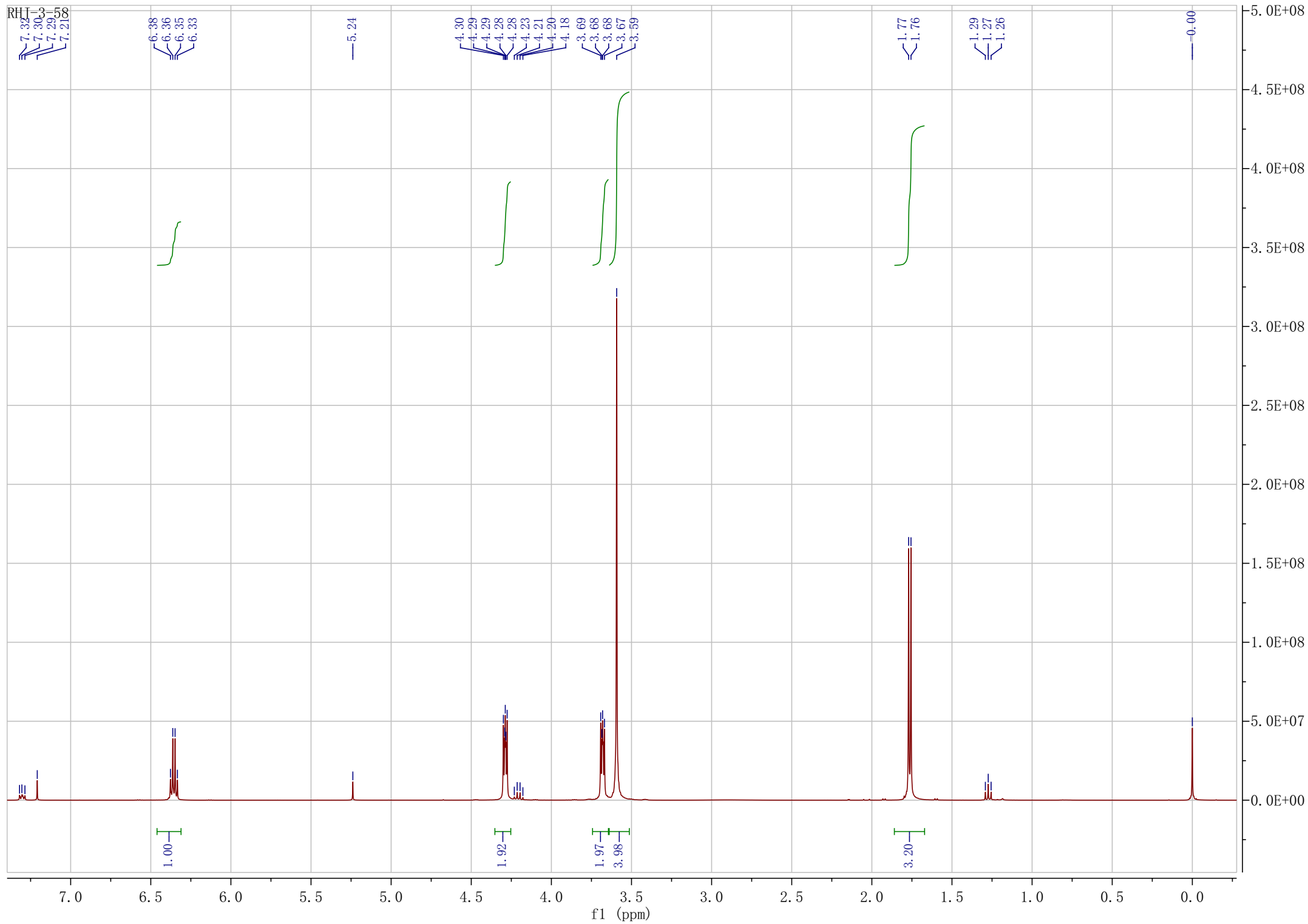

Supplement: Supplementary file 1 [file molecules-28-03912-s001.zip › molecules-2330352-supplementary/HNMR data of linker intermediate compound c.pdf]

rhj-3-61

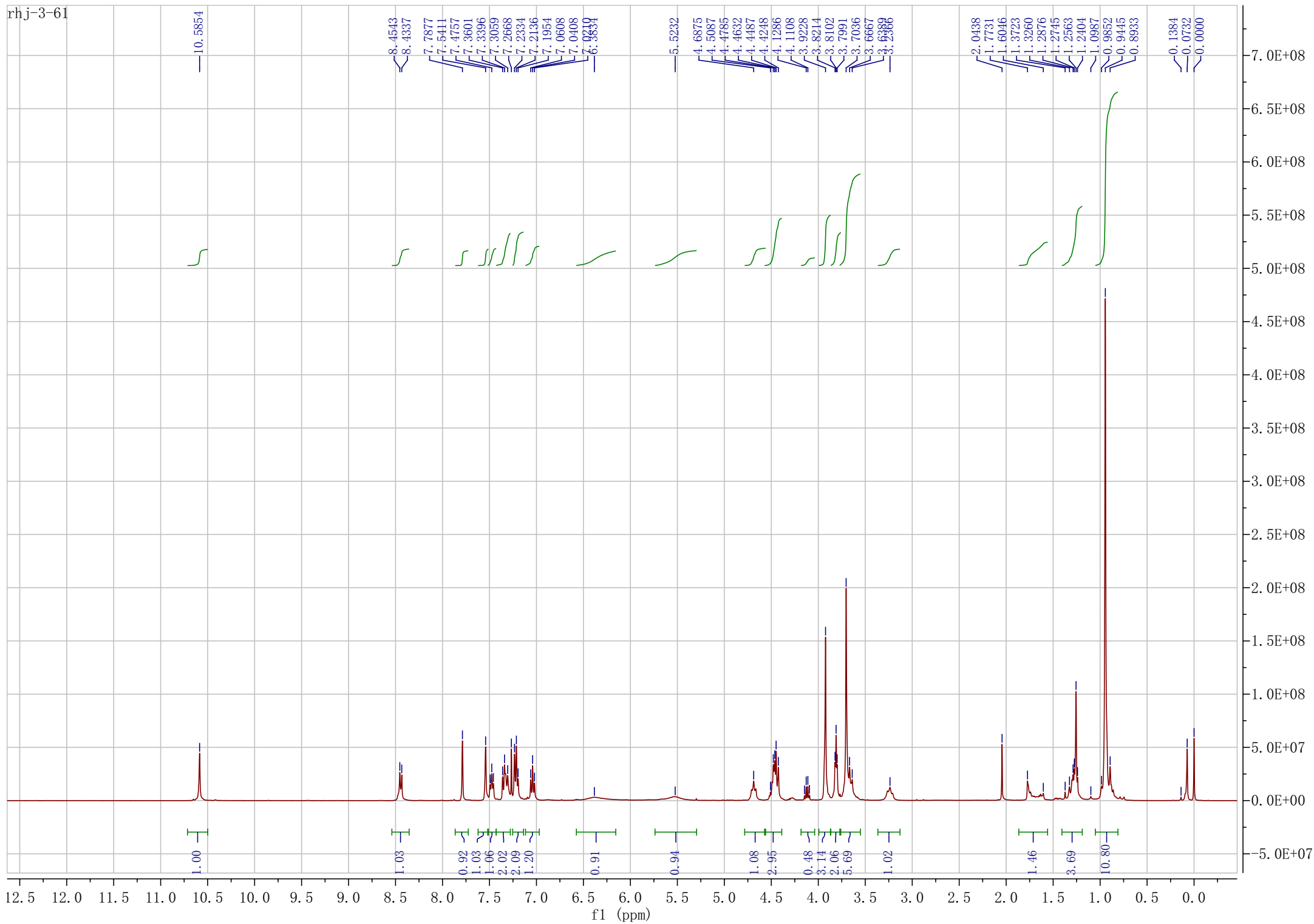

Supplement: Supplementary file 1 [file molecules-28-03912-s001.zip › molecules-2330352-supplementary/HNMR data of XR-4.pdf]
